# The Effects of Different Doses of Alfentanil and Dexmedetomidine on Prevention of Emergence Agitation in Pediatric Tonsillectomy and Adenoidectomy Surgery

**DOI:** 10.3389/fphar.2022.648802

**Published:** 2022-02-02

**Authors:** Yan-zhuo Zhang, Xiong-li Wei, Bin Tang, Yuan-yuan Qin, Min Ou, Xiao-hong Jiang, Yu-feng Tan, Mao-ying Ye

**Affiliations:** Department of Anesthesiology, Liuzhou Workers Hospital/The Fourth Affiliated Hospital of Guangxi Medical University, Liuzhou, China

**Keywords:** emergence agitation, tonsillectomy, adenoidectomy, alfentanil, dexmedetomidine

## Abstract

**Background:** Emergence agitation (EA) is a common problem often observed in children after sevoflurane anesthesia, which can be prevented by dexmedetomidine and alfentanil. This study aims to compare the effectiveness of dexmedetomidine alone and with different doses of alfentanil in preventing EA in children under sevoflurane anesthesia.

**Materials and Methods:** In a double-blind trial, 80 children (ASA I or II, 3–7 years old) undergoing tonsillectomy alone and adenotonsillectomy with sevoflurane anesthesia were randomly assigned into four groups: the control group, dexmedetomidine (DEX) group, dexmedetomidine plus 10 μg/kg alfentanil group (DEX + Alf1), and dexmedetomidine plus 20 μg/kg alfentanil group (DEX + ALf2). The incidence of EA was assessed with the Aono’s scale, and the severity of EA was evaluated with the Pediatric Anesthesia Emergence Delirium (PAED) scale. The time of tracheal extubation and time of wake were recorded. Postoperative pain and complications such as nausea and vomiting, cough, laryngospasm, and bradycardia were recorded.

**Results:** The incidence of EA was 50% in the control group, 25% in the DEX group, and 5% in the DEX + Alf1 group, and it never happened in the DEX + Alf2 group. The Aono’s scale, the PAED scale, and the FLACC scale in the control group and the DEX group were significantly more than those in the DEX + Alf1 group and the DEX + Alf2 group after the tracheal extubation (*p* < 0.05). The time of tracheal extubation of the control group and the DEX group were significantly shorter than those in the DEX + Alf1 group and the DEX + Alf2 group (*p* < 0.05). The awakening time of the DEX + Alf2 group is significantly longer than those in other groups (*p* < 0.05). The case of postoperative nausea and vomiting in the DEX + Alf1 group was fewer than those in the other groups (*p* < 0.05). And, the cases of cough and laryngospasm and bronchospasm in the DEX + Alf1 group and the DEX + Alf2 group were significantly less than those in the control group and the DEX group after the tracheal extubation (*p* < 0.05).

**Conclusion:** The combined administration of alfentanil and dexmedetomidine can reduce EA in children undergoing tonsillectomy alone and adenotonsillectomy with sevoflurane anesthesia. Dexmedetomidine plus 10 μg/kg alfentanil seems to be more appropriate than other dose combinations as it reduced EA and postoperative nausea and vomiting but did not prolong the time to awake.

## Introduction/Background

Sevoflurane is a popular anesthetic for children used worldwide because of its low pungency, rapid onset, and fast recovery properties. However, it is associated with a higher incidence of emergence agitation (EA) (as high as 80%) (Sato et al., 2010). EA in children is usually a short-lived phenomenon with no after-effect. And, it is potentially dangerous because it can make the children fall out of bed and remove the surgical dressings and intravenous catheters, which increased the extra medical cost (Dahmani et al., 2014; Driscoll et al., 2017; Choi et al., 2018; Abbas et al., 2019).

Pharmacological prophylactic interventions were a good method to prevent EA. Some research studies showed that propofol (Jalili et al., 2019), α_2_-agonist (Tsiotou et al., 2018; Tan et al., 2019), and µ-opioid agonists (Choi et al., 2011; Tan et al., 2016) were effective in preventing EA. Dexmedetomidine (DEX), a selective α_2_-adrenoceptor agonist, significantly reduces the incidence of EA in children after sevoflurane anesthesia (Bilgen et al., 2014; Yang et al., 2020). Sato M (Sato et al., 2010) found that intravenous 0.3 μg/kg DEX after induction of anesthesia reduced EA from 68 to 24% under sevoflurane anesthesia. Alfentanil, µ-opioid receptor agonists, can decrease the incidence of EA in children after sevoflurane anesthesia (Bilgen et al., 2014). Kim et al. (2009) showed that administration of alfentanil at the dose of 10 μg/kg and 20 μg/kg after induction of anesthesia reduced EA in children after sevoflurane anesthesia from 71 to 34%. Choi et al. (2016) revealed that 10 μg/kg alfentanil can reduce the EA from 64 to 32% in children receiving sevoflurane anesthesia. Even if both DEX and alfentanil were associated with reducing the incidence of the unsettling behavior after surgery, up to one in four children may present negative behaviors after the awakening of anesthesia.

The sedation caused by DEX with minimal respiratory depression made it a safer option when used in combination with opioids. We hypothesized that alfentanil and DEX have a synergistic effect, and the co-administration of DEX and alfentanil can decrease the EA to a satisfactory degree that is lower than using one drug alone in children after sevoflurane anesthesia.

In this study, we aimed to evaluate the effects of DEX alone and the co-administration of DEX and two different doses of alfentanil to prevent the unsettling behavior after sevoflurane anesthesia in children undergoing tonsillectomy and adenoidectomy.

## Materials and Methods

The study was approved by the institutional ethics committee and the Chinese clinical trial registry with written informed consent (the ethics number: ChiCTR-2000040530). After signing the informed consent with the parents of all children, 89 children aged between 3 and 7 years old with an ASA physical status I or II, who were scheduled for either a tonsillectomy alone or both an adenoidectomy and tonsillectomy, were enrolled in this prospective, randomized, double-blind, controlled study. The inclusion criteria were the children with tonsils enlargement more than II degrees, repeated infection of tonsils, or snoring. The excluded criteria were the children with asthma, cardiac disease, abnormal upper airway, obstructive sleep apnea syndrome (OSAS), developmental delay, or a history of the upper respiratory tract infection in the preceding 4 weeks.

The children were randomly divided into four groups according to the random number table: The children in the control group received normal saline intravenously for 10 mins from the induction; the children in the DEX group were given intravenously 0.4 μg/kg dexmedetomidine for 10 mins from the induction; the children in the DEX + Alf1 group were administered intravenously with 0.4 μg/kg dexmedetomidine for 10 mins from the induction and alfentanil (10 μg/kg) at the induction of anesthesia; the children in the DEX + Alf2 group were administered intravenously with 0.4 μg/kg dexmedetomidine for 10 minutes from the induction and alfentanil (20 μg/kg) at the induction of anesthesia.

The primary outcome of our study is if DEX and DEX-added alfentanil can decrease the degree of EA. The second outcome of our study is which combination of drugs inhibits EA the best and has the least side effects.

In the operating room, we monitored the children with noninvasive blood pressure (NIBP), electrocardiography (ECG), pulse oximetry (SpO_2_), and the bispectral index (BIS). The children were inducted with 3% sevoflurane with 5 L/min oxygen, lidocaine 1 mg/kg, propofol 2–2.5 mg/kg, atracurium 0.3 mg/kg, and study drugs which were prepared with another anesthesiologist. The anesthesiologist responsible for anesthesia and observation did not know which drug it was. 3 min later, we performed the tracheal intubation. After the induction of anesthesia, we intravenously administrated 2 mg/kg tramadol and 0.1 mg/kg dexamethasone for postoperative analgesia and preventing nausea and vomiting after surgery. We maintained the anesthesia with 2–3% sevoflurane mixed in 2 L/min 50% oxygen. The concentration of sevoflurane was adjusted so that the BIS value is in the range of 40–60. During the surgery, we injected 3 ml 0.5% lidocaine and 1:200,000 epinephrine mixture into the mucosa surrounding each tonsillar fossa for local anesthesia and vasoconstriction. The mean blood pressure (MAP), heart rate (HR), and SpO_2_ were recorded on arrival in the operating room (baseline), intubation time (T0), and 1 min (T1), 5 min (T5), 10 min (T10), 20 min (T20), and 30 min (T30) after intubation and at tracheal extubation (E0), 5 min after tracheal extubation (E5) and 10 min after tracheal extubation (E10). Tracheal extubation was performed when the patients breathed spontaneously, moved, and coughed.

The time of anesthesia was defined from the administration of DEX or saline until the tracheal tube was extubated. The time of tracheal extubation was defined from the end of the surgery and the discontinuation of sevoflurane until the tracheal tube was extubated. The time of awakening was defined from the end of the surgery and discontinuation of sevoflurane until the children acted on command. The incidence of EA was rated on the following Aono’s (Aono et al., 1997) four-point scale: 1 = calm; 2 = not calm but could be easily consoled; 3 = moderately agitated or restless and not easily calmed; and 4 = combative, excited or disoriented, and thrashing around. Scores of one and two were considered nonproblematic behavior, and scores of three and four were considered EA. The severity of EA was evaluated with the Pediatric Anesthesia Emergence Delirium (PAED) scale (Sikich and Lerman, 2004), which consists of five items: 1) the child makes eye contact with the caregiver, 2) the child shows purposeful actions, 3) the child is aware of his or her surroundings, 4) the child is restless, and 5) the child is inconsolable. Items 1–3 are scored as follows: 4 = not at all, 3 = just a little, 2 = quite a bit, 1 = very much, and 0 = extremely. Items 4 and 5 are scored as follows: 0 = not at all, 1 = just a little, 2 = quite a bit, 3 = very much, and 4 = extremely. When the comprehensive score was greater than 15, we considered that severe agitation appeared. Postoperative pain was assessed with the Face, Legs, Activity, Cry, Consolability scale (FLACC) (Redmann et al., 2017) at 10-min intervals from admission in the PACU for 30 min. When the FLACC scale of children was more than 4, 5 μg/kg alfentanil was administered intravenously. The incidence and severity of EA and pain were measured at E0, E5, and E10. The adverse reactions after surgery such as nausea and vomiting, cough, laryngospasm and bronchospasm, and bradycardia were recorded at E0, E5, and E10.

Statistical analyses were performed with SPSS 23.0. Demographic data such as the age, gender, weight, type of surgery, and duration of anesthesia and surgery were compared with unpaired Student’s t-tests. Differences in the incidence of EA and severe EA among the groups were analyzed using a χ^2^ test with the Fisher’s exact test correction compared in the three timepoints. Intra and postoperative hemodynamic and respiratory variables in the same subjects were compared with the Bonferroni test after repeated measures of analysis of variance. *p* < 0.05 was considered to be statistically significant.

## Results

A total of 89 children were enrolled in our study, and out of them, 9 children were excluded. Two children with asthma, 1 child with abnormal upper airway, two children with OSAS, and 4 children having a history of upper respiratory tract infection were excluded. In total, 80 children finished this study, with 20 children in each group. The demographic data such as the age, gender, surgery type, weight, duration of surgery, and anesthesia showed no significant differences among the four groups (*p* > 0.05) (Table 1).

**TABLE 1:** Demographic and surgical characteristics (*n* = 20).

	Control	DEX	DEX + Alf1	DEX + Alf2
Age (year)	4.53 ± 1.32	4.81 ± 1.09	5.13 ± 1.29	5.11 ± 1.23
Gender (M/F)	10/10	12/8	11/9	9/11
Weight (kg)	21.35 ± 9.69	21.60 ± 5.12	23.15 ± 9.31	22.69 ± 9.83
Tonsillectomy/Adenotonsillectomy	2/18	1/19	1/19	2/18
Anesthesia duration (min)	61.01 ± 11.38	62.01 ± 11.99	65.99 ± 10.87	66.01 ± 12.03
Surgery duration (min)	37.02 ± 5.80	38.04 ± 6.01	37.16 ± 5.76	37.48 ± 6.71

DEX, dexmedetomidine; Alf, alfentanil; M, male; F, female.

DEX alone treatment reduced the occurrence rate of EA from 50% in the control group to 25% in the DEX group. Co-administered 10 μg/kg alfentanil further decreased the incidence to 5% in the DEX + Alf1 group, and co-administered 20 μg/kg alfentanil decreased the incidence to 0% in the DEX + Alf2 group, respectively (Table 2). There was no difference in the time of tracheal extubation between the DEX group and the control group (*p* > 0.05), while the time of tracheal extubation was prolonged by administered alfentanil from 12.08 ± 3.69 min in the DEX group to 15.24 ± 4.68 min in the DEX + Alf1 group and 16.06 ± 4.76 min in the DEX + Alf2 group (*p* < 0.05), respectively. There was no difference in the time of tracheal extubation between the DEX + Alf1 group and the DEX + Alf2 group (*p* > 0.05). The time of awakening in the DEX + Alf two group was significantly longer than those in the other groups (*p* < 0.05) (Table 2).

**TABLE 2:** EA and the time of extubation and awaken (mean ± SD, n = 20).

	Control	DEX	DEX + Alf1	DEX + Alf2
Emergence agitation	10 (50%)	5 (25%)	1 (5%) *	0 (0) *
Time of extubation (min)	11.15 ± 3.49	12.08 ± 3.69	15.24 ± 4.68*^#^	16.06 ± 4.76*^#^
Time of awake (min)	14.95 ± 3.57	14.86 ± 3.89	15.61 ± 4.59	19.25 ± 4.38*^#Δ^

*p < 0.05, Significant difference compared with the control group; #*p* < 0.05, Significant difference compared with the DEX, group; Δ*p* < 0.05, Significant difference compared with the DEX + Alf1 group; DEX, dexmedetomidine; Alf, alfentanil.

The Aono’s scores and the scale of PAED in the DEX group, the DEX + Alf1 group, and the DEX + Alf2 group were significantly less than those in the control group at E0, E5, and E10 (*p* < 0.05). The Aono’s scores and the scale of PAED in the DEX + Alf1 group and the DEX + Alf2 group were significantly less than those in the DEX group at E0, E5, and E10 (*p* < 0.05) (Table 3) (Table 4). There was no severe EA in the DEX + Alf1 group and the DEX + Alf2 group. The severe EA in the DEX group, the DEX + Alf1 group, and the DEX + Alf2 group were significantly lower than that in the control group at E0, E5, and E10 (*p* < 0.05). The severe EA in the DEX + Alf1 group and the DEX + Alf2 group were significantly lower than that in the DEX group at E0, E5, and E10 (*p* < 0.05) (Table 5).

**TABLE 3:** Aono’S Four-Point Scale of different groups (mean ± SD, *n* = 20).

	Control	DEX	DEX + Alf1	DEX + Alf2
E0	3.20 ± 0.77	2.20 ± 0.41*	1.50 ± 0.51*^#^	1.30 ± 0.47*^#^
E5	2.95 ± 0.60	1.90 ± 0.55*	1.40 ± 0.50*^#^	1.20 ± 0.41*^#^
E10	2.70 ± 0.47	1.70 ± 0.66*	1.10 ± 0.31*^#^	1.00 ± 0.00*^#^

*p < 0.05, Significant difference compared with the control group; #*p* < 0.05, Significant difference compared with the DEX, group; DEX, dexmedetomidine; Alf, alfentanil.

**TABLE 4:** Scale of PAED in the different groups (mean ± SD, *n* = 20).

	Control	DEX	DEX + Alf1	DEX + Alf2
E0	12.49 ± 2.60	12.07 ± 2.13*	10.05 ± 1.72*^#^	10.11 ± 1.68*^#^
E5	10.29 ± 4.48	8.12 ± 4.15*	6.59 ± 2.54*^#^	6.90 ± 3.69*^#^
E10	7.65 ± 4.08	5.89 ± 3.46*	4.33 ± 2.31*^#^	4.24 ± 3.48*^#^

*p < 0.05, Significant difference compared with the control group; #*p* < 0.05, Significant difference compared with the DEX group; DEX, dexmedetomidine; Alf, alfentanil.

**TABLE 5:** Case of patients who suffered severe agitation (*n* = 20).

	Control	DEX	DEX + Alf1	DEX + Alf2
E0	5	3*	0*^#^	0*^#^
E5	3	2*	0*^#^	0*^#^
E10	2	1*	0*^#^	0*^#^

*p < 0.05, Significant difference compared with the control group; #*p* < 0.05, Significant difference compared with the DEX, group; DEX, dexmedetomidine; Alf, alfentanil.

The FLACC scale and the case of rescue alfentanil in the DEX group, the DEX + Alf1 group, and the DEX + Alf2 group were significantly less than those in the control group (*p* < 0.05). The FLACC scale and the case of rescue alfentanil in the DEX + Alf1 group and the DEX + Alf2 group were significantly less than those in the DEX group (*p* < 0.05). The cases of nausea and vomiting in the DEX group, the DEX + Alf1 group, and the DEX + Alf2 group were significantly lower than those in the control group (*p* < 0.05). And, the case of nausea and vomiting in the DEX + Alf1 group was significantly lower than those in the DEX group and the DEX + Alf2 group (*p* < 0.05) (Table 6). Furthermore, DEX plus 10 μg/kg alfentanil seems to be more appropriate than other dose combinations because it has a lower incidence of postoperative nausea and vomiting and shorter awakening time. Administration of alfentanil at the doses of 10 μg/kg and 20 μg/kg with DEX significantly reduced cases of cough and laryngospasm and bronchospasm (*p* < 0.05) (Table 6)

**TABLE 6:** Pain score and adverse reaction in different groups (mean ± SD, *n* = 20).

	Control	DEX	DEX + Alf1	DEX + Alf2
FLACC scale	5.20 ± 2.25	3.20 ± 1.03*	1.60 ± 1.27*^#^	1.40 ± 1.35*^#^
Case of rescue alfentanil	15	7*	3*^#^	2*^#^
Case of nausea and vomiting	4	2*	0*^#^	1^*Δ^
Case of cough	5	2*	0*^#^	0*^#^
Case of laryngospasm or bronchospasm	3	1*	0*^#^	0*^#^
Case of bradycardia	2	1	1	2

*p < 0.05, Significant difference compared with the control group; #*p* < 0.05, Significant difference compared with the DEX group; Δ*p*<0.05, Significant difference compared with the DEX + Alf1 group; DEX, dexmedetomidine; Alf, alfentanil.

**FIGURE 1:**
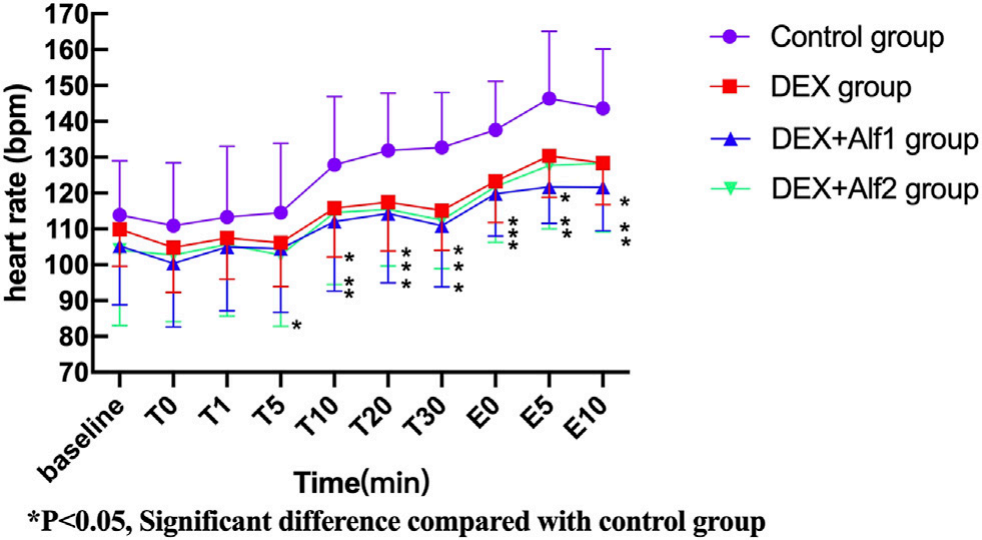
Heart rate of patients during anesthesia among groups. *p ˂ 0.05, Significant difference compared with the control group.

**FIGURE 2:**
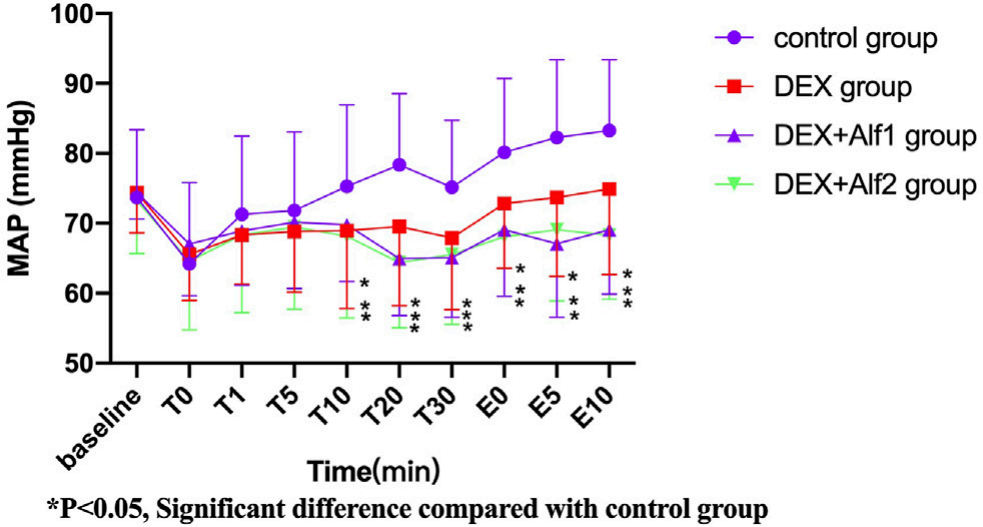
MAP of patients during anesthesia among groups. *p ˂ 0.05, Significant difference compared with the control group.

During the late stage of surgery and the period of extubation, the HR and the MAP increased gradually; DEX alone and a combination of alfentanil at the doses of 10 μg/kg and 20 μg/kg significantly inhibited the increase of the HR and the MAP (*p* < 0.05). The HR and the MAP in the DEX group, the DEX + Alf1 group, and the DEX + Alf2 group were significantly less than that in the control group at T10, T20, T30, E0, E5, and E10 (*p* < 0.05) (Figure 1 and Figure 2).

## Discussion

This study demonstrated that intravenous administration of DEX 0.4 μg/kg alone or combined with intravenous different doses of alfentanil from induction of anesthesia could reduce EA in children with sevoflurane anesthesia undergoing adenotonsillectomy surgery. In our study, the Aono’s score and the PAED scale of the DEX group, the DEX + Alf1 group, and the DEX + Alf2 group were significantly less than that of the control group. The Aono’s score and the PAED scale of the DEX + Alf1 group and the DEX + Alf2 group were significantly less than that of the DEX group; therefore, co-administration of DEX and alfentanil has a better effect to prevent EA than DEX alone. And, the time of awakening in the DEX + Alf2 group was longer than that in the DEX + Alf1 group, and the case of nausea and vomiting was more in the DEX + Alf2 group than that in the DEX + Alf1 group significantly. So intravenous administration of 0.4 μg/kg DEX with 10 μg/kg alfentanil was the appropriate dose because it can prevent EA after sevoflurane anesthesia, do not prolong the awakening time, and do not increase postoperative nausea and vomiting.

EA is a common phenomenon with children after sevoflurane anesthesia. A lot of risk factors may be considered during the development of EA, for example, pain, age, different types of surgery and inhaled anesthetics with fast emergence, and anesthetic techniques such as sevoflurane (Choi et al., 2019). In some research studies, the prophylactic use of analgesics successfully reduced EA after sevoflurane anesthesia, showed that pain may be one of the reasons for EA (Lynch et al., 1998; Kosar et al., 2014). On the other hand, post-anesthetic EA has been observed when the pain was effectively controlled (Weldon et al., 2004) or in the absence of pain (Cravero et al., 2000). In our study, The FLACC scale and the cases of rescue alfentanil in the DEX group, the DEX + Alf1 group, and the DEX + Alf2 group were significantly less than those in the control group. The differences of the FLACC scale and the Anno’s scale and the PAED scale between the groups were similar. Then, in our study, the result showed that pain is one of the major reasons for post-anesthetic EA. Children aged less than or equal to 7 years old (preschool children) are more likely to undergo EA (Przybylo et al., 2003). The patients have emergence delirium and not pain, maybe related to the inhaled anesthetics with fast emergence and anesthetic techniques as sevoflurane, the age, and the type of the surgery. In this study, we enrolled 3–7-year-old children who were more likely to suffer from EA, and in our study, the incidence of EA is 50% which is similar to the 44% in Przybylo’s research (Przybylo et al., 2003) at roughly the same age children. There is some relationship between EA and the type of surgery such as head and neck surgery, tonsils, thyroid, and middle ear surgery (Przybylo et al., 2003; Sato et al., 2010). In our study, the children we enrolled underwent tonsillectomy and adenoidectomy surgery. The age and the type of surgery subject the children at a high risk of EA.

The mechanism of DEX prevention of EA for children undergoing tonsillectomy and adenoidectomy may have sedative, analgesic, and anxiolytic properties (Olutoye et al., 2010; Zhang et al., 2019). Meanwhile, both a single dose administered and continuous intravenous infusion of DEX showed that can reduce EA after sevoflurane anesthesia in children (Yang et al., 2020). In our study, the continuous infusion of DEX for 10 minutes during induction decreased EA from 50 to 25% without prolonging awakening time. Kim found that the 50% effective dose and 95% effective dose of DEX to prevent EA in children undergoing tonsillectomy or adenoidectomy after desflurane anesthesia is 0.25 μg/kg or 0.38 μg/kg (Kim et al., 2015). And, the 0.3–0.5 μg/kg bolus dose of DEX has no hemodynamic effects in children (Guler et al., 2005; Ibacache et al., 2015). Although many research studies showed that DEX could be safely used in children, a recent study found that DEX caused some hemodynamic changes in children (Jooste et al., 2010). In our study, we choose a 0.4 μg/kg dose of DEX; it has a good effect of preventing EA and does not show any side effects.

Several research studies demonstrated that intravenous alfentanil 10 μg/kg and 20 μg/kg could decrease the incidence of EA (Aono et al., 1997; Kim et al., 2009; Choi et al., 2016). In our study, we selected these two doses combined with DEX. The mechanism of alfentanil to prevent EA after sevoflurane anesthesia may be related to its analgesic and slightly sedative effect (Choi et al., 2016). In our study, the difference of the FLACC scale and Anno’s scale and PAED scale between the groups was similar. This supported the analgesic effect of alfentanil to prevent EA. One of the side effects of opioids is postoperative nausea and vomiting. In our study, although we use the prophylactic dexamethasone, the case of nausea and vomiting in the DEX + Alf2 group was more than that in the DEX + Alf1 group. Dexamethasone can decrease postoperative edema and improve subsequent oral take after tonsillectomy because it has anti-inflammatory effects (Yiu et al., 2017).

Alfentanil can depress the respiratory depression; DEX also has minimal effect on depressing the respiratory depression. In our study, the children had not experienced respiratory depression during extubation and in the PACU. And, the children did not show hypoxemia when they came back to the ward. The MAP was stable during extubation and in the PACU in the DEX group, the DEX + Alf1 group, and the DEX + Alf2 group. The sedative effects of DEX and alfentanil make the patients’ heart rate to not increase very high. The combination of DEX and alfentanil reduced the case of nausea and vomiting, cough, laryngospasm, and bronchospasm and did not increase the case of bradycardia after surgery. Thus, the combination of DEX and alfentanil has the synergistic effect to prevent EA after sevoflurane anesthesia and has no severe side effects.

## Conclusion

Co-administration of alfentanil and DEX can reduce EA in children after sevoflurane anesthesia. DEX plus 10 μg/kg alfentanil seems to be more appropriate than other dose combinations with 20 μg/kg alfentanil as it reduced EA and postoperative nausea and vomiting but did not prolong the time to awake.

## Data Availability

The raw data supporting the conclusion of this article will be made available by the authors, without undue reservation.
